# Folate receptor-targeted molecular imaging improves identification of malignancy during pulmonary resection: a case report

**DOI:** 10.1186/s13019-017-0664-7

**Published:** 2017-12-04

**Authors:** Jarrod D. Predina, Andrew Newton, Courtney Connolly, Sunil Singhal

**Affiliations:** 1 Center for Precision Surgery, Perelman School of Medicine at the University of Pennsylvania, Philadelphia, USA; 2 Division of Thoracic Surgery, Department of Surgery, Perelman School of Medicine at the University of Pennsylvania, 6 White building; 3400 Spruce St, Philadelphia, PA 19104 USA; 3 Department of Surgery, Perelman School of Medicine at the University of Pennsylvania, Philadelphia, USA

**Keywords:** Pulmonary adenocarcinoma, Surgery, Intraoperative imaging, Folate receptor alpha

## Abstract

**Background:**

During minimally invasive pulmonary resection, both limited visualization and tactile feedback can make localization of pulmonary nodules and assessment for synchronous disease challenging. Intraoperative molecular imaging is an emerging technology that can enhance a surgeon’s ability to detect cancers at the time of resection.

**Case presentation:**

In this report, we describe the application of a folate receptor-targeted, near infrared optical contrast agent (OTL38) for the detection of an invasive pulmonary adenocarcinoma. During molecular imaging, an otherwise undetectable synchronous nodule was also identified. This finding resulted in intraoperative upstaging and operative plan modifications.

**Conclusion:**

This report marks the first successful utilization of a targeted, near infrared intraoperative molecular imaging probe useful for thoracic malignancies. This rapidly evolving technology may enhance the surgeon’s ability to perform a number of oncologic procedures including tumor localization, margin assessment and intraoperative staging.

**Electronic supplementary material:**

The online version of this article (10.1186/s13019-017-0664-7) contains supplementary material, which is available to authorized users.

## Background

During minimally invasive pulmonary resection, both limited visualization and tactile feedback can make localization of pulmonary nodules and synchronous disease challenging. Current techniques to improve intraoperative detection include intraoperative ultrasound, radionucleotide imaging, and CT-guided or spiral wire localization [1]. There are, however, challenges associated with these approaches, such as the requirement of prior knowledge regarding nodule location and the potential for patient morbidity [1]. Several groups have proposed utilization of targeted, near-infrared (NIR) fluorescent contrast agents for real-time intraoperative cancer detection; however, this has not been successfully executed for thoracic neoplasms [2]. In this report, we describe the first human experience utilizing targeted, NIR intraoperative imaging with the folate receptor alpha (FRα) targeted optical contrast agent (OTL38) to localize known pulmonary nodules and identify synchronous disease.

## Case Presentation

**Fig. 1:**
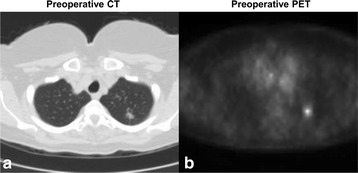
Preoperative CT (**a**) and PET (**b**) revealing an FDG-avid nodule in the LUL

**Fig. 2:**
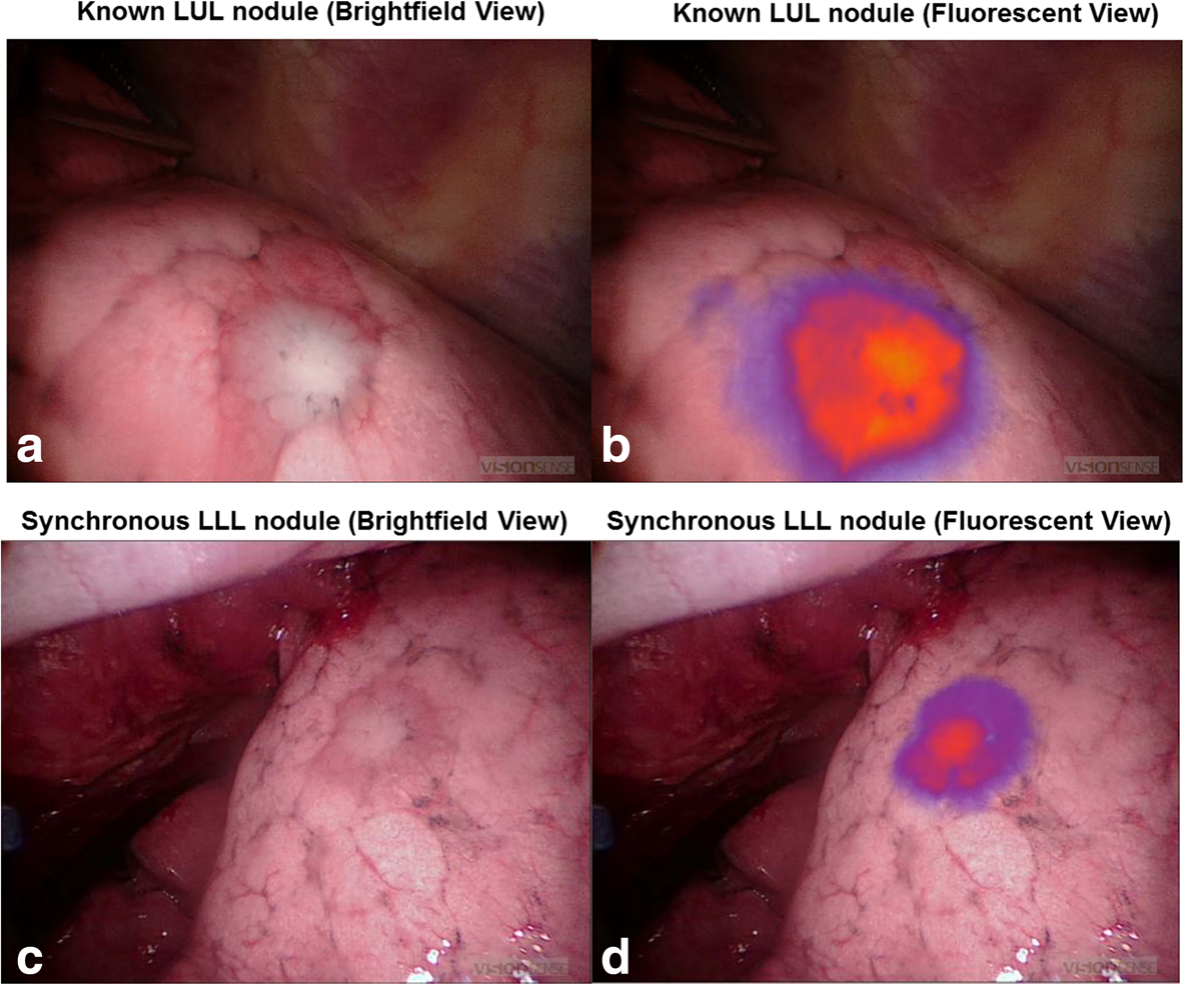
Folate receptor-targeted molecular imaging improved the surgeons’ ability to identify malignant pulmonary nodules. The preoperatively identified left upper lobe pulmonary nodule displayed high fluorescence levels (**a** and **b**). In the left lower lobe, molecular imaging identified an additional malignant pulmonary nodule (**c** and **d**) that was not identified on preoperative imaging nor by traditional intraoperative inspection techniques


A 72-year-old female with a 60-pack-year smoking history presented with an incidentally identified 2·3 cm solitary pulmonary nodule of the left upper lobe. Preoperative PET-CT confirmed FDG uptake, no suspicious lymphadenopathy or additional pulmonary pathology was observed (Fig. 1a, b). Four hours prior to tumor resection, OTL38 (0.025 mg/kg) was delivered intravenously. At the time of surgery, the preoperatively identified left upper lobe nodule displayed high levels of fluorescence (Fig. 2a, b). In addition to the known left upper lobe nodule, an additional suspicious area of fluorescence was noted in the left lower lobe **(**Fig. 2c, d **,** and Additional file 1: Video). This second area was not concerning on preoperative imaging and displayed no other obvious visual or palpable irregularities intraoperatively. The identified synchronous left lower lobe lesion was wedge resected using real-time fluorescence guidance. Frozen section analysis revealed a 0·4 cm invasive pulmonary adenocarcinoma, and final pathology confirmed folate receptor-alpha expression. Due to identification of this synchronous adenocarcinoma, the left upper lobe nodule wedge resected rather than removed by lobectomy as originally planned. In addition to intraoperative plan modification, identification of the occult left lower lobe adenocarcinoma upstaged the disease and the subject thus received systemic platinum-based chemotherapy following resection. After one year of follow-up, the patient has had no evidence of disease recurrence or drug toxicity.

Additional file 1:Intraoperative molecular imaging allowed for identification of an additional malignant pulmonary nodule in the left lower lobe. Identification of this synchronous malignancy upstaged the patient, modified operative planning, and altered postoperative adjuvant course. (MP4 49087 kb)


## Discussion

Intraoperative molecular imaging, also commonly referred to as fluorescence guided surgery, is a rapidly evolving technique that can enhance the surgeon’s ability to perform a number of intraoperative tasks including nodule localization, metastasis evaluation, and margin assessment. In this report, we demonstrate utility of NIR molecular imaging using the FRα-targeted contrast agent, OTL38.

OTL38 (chemical formula: C_61_H_63_N_9_Na_4_O_17_S_4_ (Tetrasodium salt); molecular weight: 1414.42 Da) is a folate analogue conjugated to the NIR fluorescent dye, S0456 [3] The FRα is an attractive target given known expression in many malignancies, including pulmonary adenocarcinomas [4]. OTL38 exploits highly specific FRα-targeted binding which has similarly observed with the visual contrast agent, EC17 (folate-FITC) [5]. Unlike EC17, folate is linked to a NIR fluorophore in OTL38 and emits in the NIR range (λ_ex_ 774 and λ_em_ 795) [6]. NIR imaging probes have several advantages over visual range probes; most notably, less autofluorescence and superior depth of penetration.

This report highlights several important lessons involving molecular imaging with OTL38. First, we found molecular imaging with OTL38 feasible. To elaborate, OTL38 delivery occurs 3–6 h prior to resection and imaging, thus can be delivered in the preoperative holding area. Alternative agents are delivered days prior to resection [2]. Further, using a commercially available imaging system capable of NIR detection (Iridium, Visionsense, Philadelphia, PA, USA), we found that implementation of molecular imaging only added a few minutes to the case duration. Second, we found delivery of OTL38 to be safe, with no toxicity being observed. This low toxicity profile is in accordance with the data involving other targeted intraoperative optical contrast agents currently under investigation [2, 6]. Finally, OTL38 allowed the operating surgeon to identify a synchronous subcentimeter pulmonary adenocarcinoma which would otherwise have been missed. This subcentimeter sensitivity provides data suggesting that this technology may ultimately enhance oncologic resections by identification of positive/close resection margins or small synchronous cancer foci that are not detected preoperatively or intraoperatively. At this point, we are further evaluating this technology in a Phase I clinical trial (NCT02602119).

## Conclusion

This report marks the first successful utilization of a targeted, near infrared intraoperative molecular imaging probe useful for thoracic malignancies. In the future, this technology may enhance the surgeon’s ability to perform a variety of oncologic procedures including tumor localization, margin assessment and intraoperative staging.

## Abbreviations

CT: Computed tomography
FR: Folate receptor
IMI: Intraoperative molecular imaging
NIR: Near infrared
PET: Positron emission tomography
